# The Allelic Diversity of the Gibberellin Signaling Pathway Genes in *Aegilops tauschii* Coss

**DOI:** 10.3390/plants9121696

**Published:** 2020-12-02

**Authors:** Mikhail S. Bazhenov, Anastasiya G. Chernook, Nikolay P. Goncharov, Nadezhda N. Chikida, Mariya Kh. Belousova, Gennady I. Karlov, Mikhail G. Divashuk

**Affiliations:** 1 Laboratory of Applied Genomics and Crop Breeding, All-Russia Research Institute of Agricultural Biotechnology, Timiryazevskaya Street, 42, 127550 Moscow, Russia; irbis-sibri@yandex.ru (A.G.C.); karlovg@gmail.com (G.I.K.); divashuk@gmail.com (M.G.D.); 2 Wheat Genetics Laboratory, Institute of Cytology and Genetics, Siberian Branch of the Russian Academy of Sciences, Akademika Lavrentieva Avenue, 10, 630090 Novosibirsk, Russia; gonch@bionet.nsc.ru; 3 Federal Research Center N. I. Vavilov All-Russian Institute of Plant Genetic Resources (VIR), 190000 Saint Petersburg, Russia; n.chikida@vir.nw.ru; 4 Dagestan Experimental Station—The Branch of the Federal Research Center N. I. Vavilov All-Russian Institute of Plant Genetic Resources, Vavilovo Village, Derbent District, 368600 Dagestan, Russia; m.h.belousova@mail.ru; 5 Kurchatov Genomics Center–ARRIAB, All-Russia Research Institute of Agricultural Biotechnology, Timiryazevskaya Street, 42, 127550 Moscow, Russia

**Keywords:** *Rht-D1* gene, *Gid1-D*, and *Gid2-D* genes, gibberellins, reduced plant height, wheat, *Aegilops tauschii*

## Abstract

Gibberellin-insensitive reduced height genes are widely spread in modern wheat varieties, making them resistant to lodging under conditions of intensive farming. However, the limited diversity of these genes present in wheat germplasm can limit the adaptability of newly created cultivars to the changing climate. The diversity of the gibberellin signaling pathway genes involved in plant height control—*Reduced height 1* (*Rht-D1*), *Gibberellin-insensitive dwarf 1* (*Gid1-D*) and *Gibberellin-insensitive dwarf 2* (*Gid2-D*)—was studied in the diploid wild goatgrass *Aegilops tauschii* Coss., one of the ancestral species of the bread wheat (*Triticum aestivum* L.) and the donor of its D subgenome, using high-throughput sequencing. The examination of 24 *Ae. tauschii* accessions of different geographical origins revealed a large number of new alleles (haplotypes) not found in bread wheat varieties. Some of the detected polymorphisms lead to changes in the amino acid sequence of proteins. Four isoforms (amino acid sequence variants) were found for the RHT-D1 protein, and two isoforms—for the GID1 and GID2 proteins, each. An analysis of the co-occurrence frequencies of various isoforms of the three proteins showed that their combinations were not random in *Ae. tauschii*, which may indicate the functional significance of their differences. New alleles of the *Rht-D1*, *Gid1-D*, and *Gid2-D* genes are promising for introgression into bread wheat and studying their effect on plant height and adaptability.

## 1. Introduction

The introduction of the *Reduced height* (*Rht*) genes associated with insensitivity to gibberellins, plant growth hormones into new varieties caused a rapid increase in wheat productivity in the second half of the XX century [1]. Gibberellin-insensitive reduced height genes can enhance the resistance of wheat varieties to lodging, especially under conditions of high doses of nitrogen fertilizers, making them adapted to cultivation under intensive farming [2]. Currently, several dozen reduced height genes and their alleles have been described [3]. At the same time, the gibberellin-insensitive short stature trait of most modern commercial wheat varieties is provided by mutations of only two genes—*Rht-B1* and *Rht-D1*. Mutations of these genes—*Rht-B1b* (previous designation *Rht1*) and *Rht-D1b* (previous designation *Rht2*)—were originally transferred to the CIMMYT-developed varieties (International Center for the Improvement of Maize and Wheat/Centro Internacional de Mejoramiento de Maíz y Trigo), and later to European varieties from the Japanese dwarf cv. Norin 10, and ensured the success of the Green Revolution [4]. The *Rht-B1e* mutation was also widely introduced in Russian wheat cultivars [5]. Despite the known disadvantages, gibberellin-insensitive dwarfism is still preferred for wheat varieties cultivated under sufficient moisture conditions [6].

The reduced height of plants can be associated with both the impaired biosynthesis of gibberellins and the accumulation of repressors of the hormonal signal of gibberellins—the DELLA proteins, while tall plant phenotype can be associated with damage to the gibberellin deactivation enzymes or the loss of the repressive function of the DELLA proteins. The *Rht-B1* and *Rht-D1* genes encode DELLA proteins, which function as transcriptional coactivators and corepressors [7,8]. These proteins are negative regulators of the gibberellin signaling pathway. A high level of DELLA proteins in plant cells suppresses the growth of their vegetative organs, while the activation of growth by gibberellins is mediated by the degradation of these proteins [1]. The gibberellin receptor, GID1 (GIBBERELLIN-INSENSITIVE DWARF 1), plays an important role in regulating the cell level of DELLA proteins. GID1 in the presence of these hormones acquires the ability to bind to DELLA proteins, after which the GID1-DELLA complex is recognized by the F-box proteins—SLY1 (SLEEPY1) and GID2 (GIBBERELLIN-INSENSITIVE DWARF 2), which form ubiquitin ligase complex, after which DELLA-protein is ubiquitinated and subjected to proteasome degradation [9]. The *Gid1* gene (*Gibberellin-insensitive dwarf 1*) was first identified in dwarf rice mutants that did not respond to treatment with exogenous gibberellic acid by increasing the growth of experimental plants [10]. The decrease in expression of the *Gid1* gene and, as a consequence, a dwarf phenotype was revealed in a series of different plant mutants. DELLA proteins contain two main domains—the N-terminal DELLA domain, which is involved in the interaction with GID1, and the GRAS domain, which possesses transactivation, repressive, and regulatory activities [9]. Reduced height mutations of the wheat *Rht-1* gene are associated with damage or absence of the DELLA domain, which makes these proteins more stable in the cell and, accordingly, significantly reduces the growth processes stimulated by gibberellins in mutant plants [11].

In bread or common wheat (*Triticum aestivum* L.; 2*n* = 6*x* = 42, BBAADD genome), DELLA proteins are encoded by *Rht-A1*, *Rht-B1*, and *Rht-D1* genes, referring, respectively to its subgenomes A, B, and D. The *Rht-A1* gene does not have any known reduced-height mutations. The *Rht-B1* gene has the largest number of ones. Its *Rht-B1b*, *Rht-B1d*, *Rht-B1e*, and *Rht-B1p* alleles determine the semi-dwarf phenotype caused by the emergence of stop codons within a small region of the *Rht* gene, after which translation is likely to be reinitiated [12,13]. The *Rht-B1c* allele determines the extreme dwarf phenotype caused by the insertion of the retrotransposon, which leads to the occurrence of an intron in the gene and a 30-amino acid insertion in the region of the protein DELLA domain [12]. In addition, numerous agronomically important reverse mutations of the *Rht-B1c* allele with a semi-dwarf phenotype were obtained [14]. *Rht-D1* gene has a smaller number of known reduced-height mutations—the *Rht-D1b* allele (originally described as the *Rht2* gene), due to a stop codon, and the *Rht-D1c* allele that determines the extreme dwarf phenotype and represents a duplication of the *Rht-D1b* allele [15]. In addition to the alleles listed, a significant number of functionally neutral mutations have been described in the *Rht-1* genes of polyploid wheat [16]. There are also many mutations associated with an increase rather than a decrease in plant height [17]. The allelic diversity of the wheat *Gid1* and *Gid2* genes known to date is scarce [18,19]. The lack of involvement of the *Gid1* and *Gid2* genes in breeding programs for the development of commercial semi-dwarf wheat varieties could be explained by the recessiveness of their dwarfing mutations, which could be assumed based on the function of the encoded proteins, and the requirement of the simultaneous presence of recessive mutations in all homoeologous genes of allopolyploid wheat for expression of the dwarfism.

The alleles of the wheat *Rht-1* genes are known to differ in the degree of influence on plant height and other agronomically important traits, while their pleiotropic effects do not necessarily strictly correlate with each other. Thus, the *Rht-D1b* allele reduces the resistance of wheat plants to *Fusarium* head blight to a greater extent than the *Rht-B1b* allele [20], and the *Rht-B1c.23* and *Rht-B1c.26* alleles shorten the stem length only slightly more than the *Rht-B1b* allele, but at the same time significantly increase the duration of the seed dormancy period, rising the resistance of wheat to preharvest sprouting [14]. This means that the study of the polymorphism of the gibberellin signaling pathway genes can enrich the toolkit of breeders with new alleles that have unique valuable combinations of multiple phenotypic expressions.

Many studies have shown the existence of a significant allelic diversity of agronomically important genes in diploid ancestral wheat species and its wild relatives in comparison with widely cultivated tetraploid and hexaploid wheat species [21,22]. *Aegilops tauschii* Coss. (= syn. *Ae. squarrosa* L.; 2*n* = 2*x* = 14, DD genome) is one of the ancestral species of bread wheat and a donor of its D subgenome [23,24]. It also participated in the formation of many polyploid species of the genus *Aegilops* L. as a donor of their cytoplasmic genome [25]. However, during bread wheat evolution, only a handful of *Ae. tauschii* accessions from a small region hybridized with wheat leading to a narrow genetic base of the wheat D subgenome. Therefore, *Ae. tauschii* should be used more widely in bread wheat breeding.

At present, there is an intensive search for molecular polymorphisms [26,27], which could be used in wheat breeding in the future. The genetic diversity of *Ae. tauschii* has been proved to exceed significantly that of the D genome of polyploid wheat [28]. For this reason, *Ae. tauschii* is among the most promising donors of economically valuable traits for bread wheat, and, therefore, the study of diploid ancestral species of polyploid wheat is promising for the search for the new gene variants. These variants may prove to be agronomically important if transferred to widely cultivated wheat species through specially designed bridge species [29] or by producing synthetic allopolyploids [30,31].

The objective of this research is to study the allele diversity of the gibberellin signaling pathway genes—*Rht-D1*, *Gid1-D*, and *Gid2-D*—in *Ae. tauschii*.

## 2. Results

### 2.1. Rht-D1

The analysis of the nucleotide sequences of the *Rht-D1* gene, together with the promoter region, identified 7 allele variants (haplotypes) in 24 *Ae. tauschii* of different geographic origins (Table S1), none of which contained a stop codon characteristic of the *Rht-D1b* allele of bread wheat, which is functionally significant for decreasing plant height. The *Rht-D1* alleles of common wheat, which have no functional significance and control the “tall” phenotype of plants, were previously designated by numbers from 1 to 6 (*Rht-D1a_1… 6*) [16]. Later, another allele, *Rht-D1a_7* (JX993604), was also deposited in the NCBI GenBank database. We identified only two of these previously described alleles in *Ae. tauschii—Rht-D1a_5* and *Rht-D1a_7*, which turned out to be the most common among the accessions of this species studied here. The four other described here alleles turned out to be completely new, and we designated them as *Rht-D1a_8…11* (Table 1).

The fully sequenced genome of the *Ae. tauschii AL8/78* accession [21] contained one more allele variant, which we designated as *Rht-D1a_12*. The differences between alleles of *Rht-D1* are represented by 4 single nucleotide substitutions (SNS) and a multiple-nucleotide substitution (MNS) without a frameshift in the coding sequence and by 10 SNS and 2 short insertions/deletions (InDel) in the promoter and non-coding sequence. Two of the four SNS and an MNS in the coding region lead to non-synonymous codon change. The seven alleles give a total of four isoforms of the RHT-1 protein, which are designated in this work in capital letters. The letters A and B represent the most common isoforms, while the letters C and D stay for the rare ones. The correspondence between the gene alleles and protein isoforms, as well as the frequency of their occurrence among the *Ae. tauschii* accessions are presented in Table 1.

**Figure 1:**
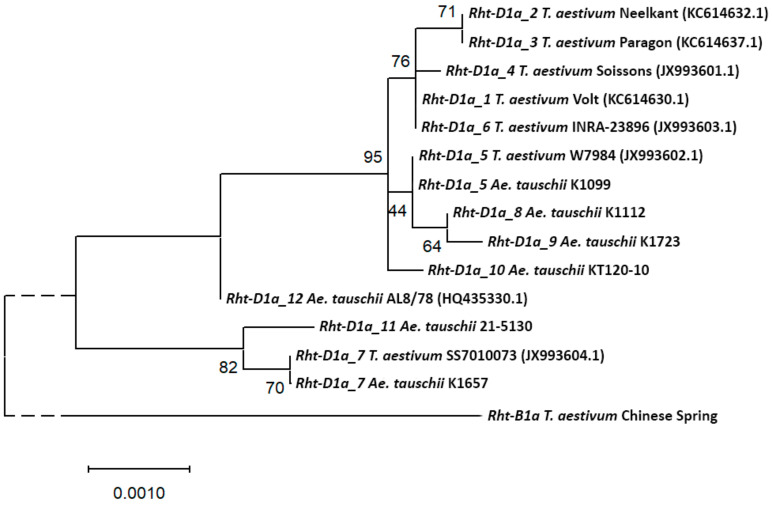
Molecular phylogenetic tree of the *Rht-D1* gene alleles of *Ae. tauschii* and *T. aestivum*, constructed by the maximum-likelihood method. After the allele name, the catalog number of the *Ae. tauschii* accession or the wheat variety in which it was detected is indicated. The GenBank accession numbers for the nucleotide sequences are given in parentheses. The percentage of trees in which associated alleles clustered together in 500 bootstrap replicates is shown next to the branches. The *Rht-B1* gene of common wheat was used as an outgroup.

Phylogenetic analysis of the *Rht-D1* alleles shows that the *Rht-D1a_12* allele, which corresponds to the D protein isoform (*Ae. tauschii* accession AL8/78), is the closest to the ancestral wild variant of the gene (Figure 1). *Rht-D1a_5*, *Rht-D1a_8*, and *Rht-D1a_9* corresponding to the isoform A of the protein form a common cluster, which occurs almost at the same time with the *Rht-D1a_10* allele, which corresponds to the isoform C the of protein. It should be noted that the isoform C of the protein is encoded by most of the known alleles of the *Rht-D1* gene of bread wheat, corresponding to a tall plant phenotype (*Rht-D1a_1… 4*, *Rht-D1a_6*). *Rht-D1a_7* and *Rht-D1a_11*, corresponding to the protein isoform B, represent a branch of the phylogenetic tree, independent of the other alleles described here.

The PROVEAN prediction of amino acid substitutions functional significance showed that only the substitution of a glycine residue for serine at position 334 (G334S), which is due to the G/A transition at position 1000 from the start codon, is expected to influence the biological activity of the protein (Table 2). This variant is found only in isoform A. At the same time, isoform A, according to PROVEAN, should be less biologically active than the others. The G334S substitution occurs in the GRAS domain of the protein and thus should correspond to the taller plant phenotype.

### 2.2. Gid1-D

The study of the *Gid1-D* gene sequences together with its promoter in *Ae. tauschii* accessions revealed 13 alleles, which we designated with lowercase letters from *a* to *m*, ranking them in descending order of population frequency (Table 3). Only single nucleotide substitutions were present between alleles within the gene sequence itself, while both SNSs and short, up to 19 nucleotides, InDels were present in the promotor. Among the four SNS, the alleles differ in the coding region; only one (G926C) leads to a non-synonymous codon change. As a result, the GID1 protein in *Ae. tauschii* is represented by only two isoforms, which we designated with capital letters A and B. The frequency of isoform A among the studied accessions is 0.75; of isoform B is 0.25.

**Figure 2:**
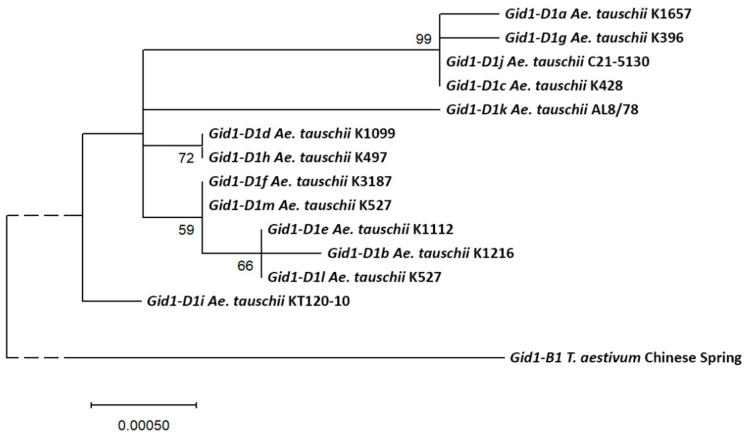
Molecular phylogenetic tree of the *Gid1-D* gene alleles of *Ae. tauschii*, constructed by the maximum-likelihood method. After the allele name, an example of the *Ae. tauschii* accession in which it was detected is given. The percentage of trees in which associated alleles clustered together in 500 bootstrap replicates is shown next to the branches. The *Gid1-B* gene of common wheat cv. Chinese Spring was used as an outgroup.

A phylogenetic analysis of the alleles of the *Gid1-D* gene showed that *Gid1-D1i*, found in the *Ae. tauschii* KT 120-10 accession from China, is the most distant from others and at the same time close to the tree root, that is, to the ancestral variant (Figure 2). It, like many other alleles, corresponds to the isoform of protein A. The *Gid1-D1b*, *e*, and *l* alleles cluster together, which indicates their common origin. They correspond to the protein isoform B. The *Gid1-D1d* allele, found in K-1099 (Azerbaijan) and K-2271 (Armenia) accessions, fully corresponds to the *Gid1-D* allele in bread wheat cv. Chinese Spring (according to the IWGSC RefSeq1.0 genome assembly). The *Gid1-D1* allele of the *Ae. tauschii* AL8/78 accession branches out separately.

The two GID1 protein isoforms differ in a single amino acid residue at position 309 of the primary structure. Isoform A contains glycine (G) at this position, while the isoform B contains alanine (A). Despite the similar chemical properties, the PROVEAN prediction indicates that this substitution is essential for the functioning of the protein, and isoform B should be less biologically active than isoform A.

### 2.3. Gid2-D

The *Gid2-D* gene, together with the sequence of its promoter, in the studied plants of *Ae. tauschii* was represented by eight alleles, which we designated with lowercase letters from *a* to *h* in accordance with their frequencies (*a*—the most frequent, *h*—the rarest) (Table 4). Three SNS in the coding region, 18-nucleotide deletion in an intron, and multiple SNS and short (1–3 nucleotides) InDels in the promotor region were detected between alleles. As in the case of the *Gid1-D* gene, the GID2 protein is represented by only two isoforms. More rare isoform B corresponds to the *Gid2-D1b* allele, and isoform A corresponds to all other alleles.

**Figure 3:**
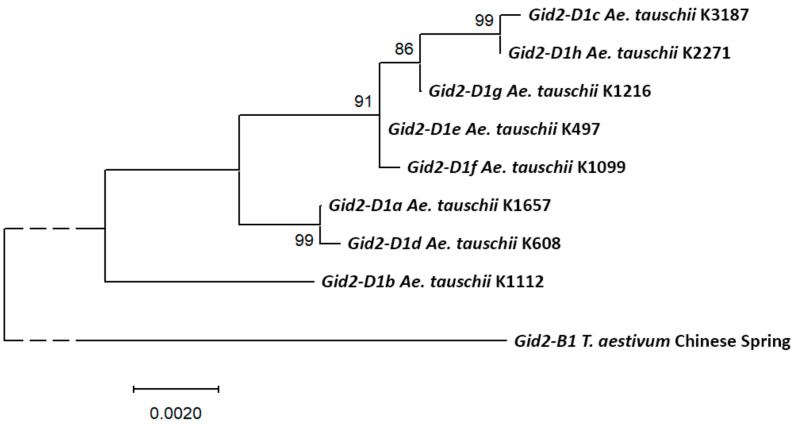
Molecular phylogenetic tree of the *Gid2-D* alleles of *Ae. tauschii*, constructed by the maximum-likelihood method. After the allele name, an example of the *Ae. tauschii* accession in which it was detected is given. The percentage of trees in which associated alleles clustered together in 500 bootstrap replicates is shown next to the branches. The *Gid2-B* gene of the cv. Chinese Spring of common wheat was used as an outgroup.

Phylogenetic analysis of alleles of the *Gid2* gene showed that *Gid2-D1b* is the closest to the ancestral form and, at the same time, differs from other alleles of the gene (Figure 3). None of the sequences of the *Gid2* gene described in *Ae. tauschii*, together with its environment, did not correspond exactly to the genomic sequence of the cv. Chinese Spring of common wheat. The *Gid2-D1c* allele is the closest to *Gid2-D1* of the cv. Chinese Spring. The *Gid2-D1e* allele was found in the AL8/78 accession, as well as in K-497 and KT 120–10.

Isoforms of the *GID2* protein differ in two consecutive amino acid residues. Isoform A contains residues of glutamic acid (E) and arginine (R) at positions 157–158, and isoform B contains residues of glycine (G) and glutamine (Q). Despite different chemical properties, PROVEAN indicates that both protein isoforms are equally functional.

### 2.4. Co-Occurrence Of Protein Isoforms

Analysis of the joint occurrence of different isoforms of three proteins of the gibberellin signaling pathway—RHT-1, GID1, and GID2 in *Ae. tauschii*, based on their genotypes, carried out using Fisher’s exact test, showed that their combinations were not random. In the presence of isoform B of RHT-1 protein (variant G of the polymorphism G334S), only isoform A of protein GID1 and only isoform A of protein GID2 are encountered. In the presence of the isoform A of the RHT-1 protein (variant S of the polymorphism G334S, presumably reducing the functionality of the GRAS domain), two isoforms of GID1 with approximately equal probability, and two isoforms of GID2 are found (Table 5).

For proteins GID1 and GID2, isoforms A are most often found in combination with each other, and both of them are most frequently combined with isoform B of the RHT-1 protein (Table 6).

One-way analysis of variance revealed no significant differences in *Ae. tauschii* plant heights differing in isoforms of RHT-D1, GID1, and GID2 proteins.

### 2.5. Data Availability

The sequences of *Rht-D1* gene were deposited to NCBI GenBank database under accession numbers MW208426—MW208432; sequences of *Gid1-D* gene—under accession numbers MW218938—MW218950; *Gid2-D* gene—under accession numbers MW218951—MW218958.

## 3. Discussion

Currently, wild-related species of crop plants are increasingly used to search for new alleles of genes that provide resistance to biotic and abiotic stresses [22], as well as genes that control other agronomically important traits. This is due to their high adaptability, great genetic diversity, and the possibility of transferring valuable alleles to crop species by distant hybridization [22,30,33].

*Ae. tauschii* is a wild self-pollinating grass, a donor of the D subgenome of bread wheat. The species has a vast natural range in central Eurasia, spreading from Turkey to western China, and is mainly found in the Caucasus and Iran along the coast of the Caspian Sea [34]. Within the entire range, *Ae. tauschii* is represented in the form of small, isolated from each other populations [35]. Populations of *Ae. tauschii* have adapted well to a variety of growing conditions, including sandy shores, rocky hills, roadside, and wet forests. *Ae. tauschii* is often found as a weed in wheat and barley fields [36]. Nowadays, the efforts of geneticists and breeders have aimed at the recruitment of the *Ae. tauschii* gene pool for the improvement of modern cultivars of bread wheat.

Morphologically, *Ae. tauschii* is traditionally divided into two subspecies—*Ae. tauschii* Coss. ssp. *tauschii* and *Ae. tauschii* Coss. ssp. *strangulata* (Eig) Tzvel. Using molecular methods, it was divided into two evolutionary lines [37]. The *tauschii* subspecies has the widest distribution and a wide variety of forms, which are traditionally grouped into four botanical varieties [38]. The *strangulata* subspecies is widespread in the southern part of the Caspian Sea and, according to some early studies, in the Transcaucasia and is distinguished by wider spikelets [34,39]. The *strangulata* subspecies is considered the most likely donor of the D subgenome of bread wheat [39]. The *tauschii* subspecies is much less studied for this reason. Therefore, we mainly studied the *tauschii* subspecies, the gene pool of which was not previously involved in the formation of the biodiversity of the D genome of hexaploid wheat. In addition, the *strangulata* subspecies is believed to be more polymorphic than the *tauschii* subspecies [40].

In this work, we studied the genes of the gibberellin hormone signaling pathway—*Rht-D1*, *Gid1-D*, and *Gid2-D*, and described new alleles for each of them (Table 1, Table 2, Table 3 and Table 4). We found seven *Rht-D1* haplotypes in *Ae. tauschii*, which give four protein isoforms differing in amino acid sequence. Moreover, all these haplotypes are not natural for bread wheat. The *Rht-D1a_5* and *Rht-D1a_7* haplotypes were previously described only in synthetic allopolyploids [16]. Most of the *Rht-D1* alleles of bread wheat, which control the tall plant phenotype, encode isoform C of the RHT-D1 protein, which was found in only one accession in our study—*Ae. tauschii* ssp. *tauschii* KT120-10 from China (Figure 1). Thus, only a small part of the diversity of the *Ae. tauschii Rht-D1* gene was transferred to bread wheat, and there is a prospect of expanding the wheat gene pool through the development and involvement of synthetic allopolyploids in modern breeding programs.

According to PROVEAN, isoform A of RHT-D1 protein should suppress stem growth to a lesser extent than isoform B. This is partially confirmed by the data of previous studies, according to which, *Ae. tauschii* plants are shorter in the eastern regions of the species range than in the western parts [34,41]. According to our data, protein isoform B is more common in the eastern regions—Pakistan, Uzbekistan, Afghanistan (Table S1). Perhaps the development of isoform B of the RHT-D1 protein is the result of plant adaptation to the sharply continental climate of this part of the range. Statistical analysis of our data on *Ae. tauschii* plant height showed no significant association of this trait with the isoforms of the RHT-D1 protein. It is possible that an adaptation of *Ae. tauschii* through the *Rht-D1* mutation is not directly related to plant height, but to other traits regulated by gibberellins, for example, the timing of anthesis (heading) or the duration of the seed dormancy. It is also possible that the expression of various *Rht-D1* alleles was compensated for by other genes—*Gid1-D* and *Gid2-D*, the alleles of which, as we have shown, are not randomly combined with the *Rht-D1* alleles (Table 5).

The *Ae. tauschii Gid1-D* gene has 13 different alleles found to encode two isoforms of the protein, designated by us with letters A and B. We found that isoform B of the RHT-D1 protein occurs only together with GID1-D isoform A, and GID1-D isoform B is found only with RHT-D1 isoform A. This joint occurrence of two proteins’ isoforms can be explained by the natural selection of their combinations. The GID1-D isoform B is encoded by a relatively young monophyletic group of alleles. According to PROVEAN, this isoform should be less functional, which means, most likely, associated with the short stature of plants. However, for the GID1-D isoforms, we were unable to establish a statistically significant relationship with plant height. Most likely, the study of the phenotypic expression of the detected alleles will become possible only through hybridological analysis. Nevertheless, the dendrogram (see Figure 2) clearly shows three clusters. The upper one is formed by the accessions from Central Asia, the middle one—from the Transcaucasia. An accession from China is located separately. This concordance of genetic and geographical divergence makes it possible to select specific accessions from specific regions to transfer the vast genetic diversity of *Ae. tauschii* into bread wheat by a limited number of interspecific crossings.

The *Gid2-D* gene showed to have eight alleles encoding two isoforms of the GID-2 protein. Isoform A corresponds to most of the identified alleles, and isoform B corresponds to the *Gid2-D1b* allele, which is closest to the ancestral variant of the gene.

The closest to the ancestral variants of the *Rht-D1* and *Gid2-D* genes are found in Armenia, Azerbaijan, and Iran. Combinations of rare alleles are observed in the same area. All this confirms the hypothesis about the center of origin of *Ae. tauschii* in Transcaucasia [38,42]. As for the identification of a *Gid1-D* allele that is close to the ancestral, in plants collected in China, this one could have been introduced there by humans as part of the *Ae. tauschii* genome, which existed as a weed in bread wheat fields. This allele persisted there due to the isolation of the Chinese populations of *Ae. tauschii* from the rest of the range. Probably, this form of *Ae. tauschii* introduced into China was a descendant of the one that once donated the D subgenome for bread wheat. This is also evidenced by the discovery of the *Rht-D1* allele encoding the protein isoform C in KT 120–10 accession from China, which is characteristic of bread wheat, but rarely occurs in the studied plants of *Ae. tauschii*.

We do not know any examples of height-reducing genes transferred from *Aegilops* species with D genome to bread wheat [43]. Therefore, our findings provide useful information for further unlocking of the genetic mechanism of agronomic trait control in wheat.

## 4. Materials and Methods

### 4.1. Plant Material

We used the collection of *Ae. tauschii*, represented by accessions of different ecological and geographical origins (Table S1). The *Ae. tauschii* accessions partially were provided by the Federal Research Center N.I. Vavilov All-Russian Institute of Plant Genetic Resources (VIR), Russia; Czech Institute of Plant Industry, Czech Republic; Kyoto University, Japan; Institute Biology of Kihara Foundation, Japan.

Data on the plant height of some *Ae. tauschii* accessions were collected during growing on the plots in the field in 2014 and in 2017 at the Dagestan Experimental Station of VIR in the process of the *Ae. tauschii* collection reproduction. The average values for the two years are shown in Table S1.

### 4.2. DNA Extraction and PCR

To isolate the DNA, the leaves of the plants growing in the greenhouse were collected into plastic microtubes, freeze-dried and crushed into a fine powder using stainless steel beads on a TissueLyser II bead mill (Qiagen, Hilden, Germany). DNA was extracted from the powder of leaves using the traditional method with a cetyltrimethylammonium-bromide-based extraction solution [44]. Two individual plants were taken for each accession; each of them represented a biological replication during sequencing.

The PCR primers were designed using the PrimerBLAST resource (NCBI) [45] and ordered for synthesis in Syntol LLC (Moscow, Russia). The primers Rht-D1-1F: 5′-TGACTTGAGATCACCTCTTGTGC-3′, Rht-D1-1R: 5′-ACACCATCATCTTGTCCTCGG-3′ (1152 b. p. product covers promotor and the forward part of the coding sequence), Rht-D1-2F: 5′-GATAGATAGAGAGGCGAGGTAGC-3′, Rht-D1-2R: 5′-TACTTACAGCGTTCAAAACTCGC-3′ (1931 b. p., coding sequence) were used to amplify the *Rht-D1* gene and part of its promotor; the primers GID1-D-F1: 5′-CTATATGCATGCGCGAGCGAAA-3′, GID1-D-R1: 5′-GGGAAGCGCCAAACAGAGGA-3′ (1174 b. p., promotor and 1-st exon), GID1-D-F2: 5′-GGGAGATCTTGCTCTTACTCCTG-3′, GID1-D-R2: 5′-CTAGAGATCACTTGCCAGAAGCC-3′ (1997 b. p., complete coding sequence), GID1-D-F5: 5′-AAGAGCCTCATCATCGTGTCG-3′, GID1-D-R5: 5′-ACCGAGAATGGATGGATCAACTTA-3′ (1095 b. p., 3′-untranslated region) were used for the *Gid1-D* gene; and the primers GID2-D-1F: ATGGAATCATGTACCGGCCAGA, GID2-D-1R: GATCACGACCAGCCGAGGAAT (1100 b. p., promotor), GID2-D-2F: TCGTCTCCTCTATCCTCGTCAAG, GID2-D-2R: GAACTGAAGGGTGTGCTCGC (1244 b. p., complete coding sequence) were used to amplify the *Gid2-D* gene. For primers design, we used the sequences of the genes from the annotated *Aegilops tauschii* AL8/78 genome assembly Aet_MR_1.0 found in the NCBI database (*Rht-D1*–LOC109733020, *Gid1-D*–LOC109741215, *Gid2-D*–LOC109786639).

The PCR mix consisted of the following components (the end concentrations are given): 1 × LR-plus buffer pH 9.3 (Sileks LLC, Moscow, Russia), 1.5 mM MgCl_2_, 0.2 mM of each dNTP, 2 µM of each primer, 0.04 U/µL LR Plus (long reading) polymerase (Sileks), 0.02 U/µL Taq-polymerase (Sileks), 4 ng/µL of matrix DNA. The volume of the PCR mix was 25 µL per tube. The PCR was conducted under the following temperature conditions: 94 °C—5 min; 36 cycles 94 °C—30 s, 58 °C—30 s, 72 °C—2 min; 72 °C—5 min.

### 4.3. Sequencing

The amplified fragments of the three genes (*Rht-D1*, *Gid1-D*, *Gid2-D*) were obtained from each of the 23 *Aegilops tauschii* accessions listed in Table S1 (excluding AL8/78) with double replication (DNA of the two plants was used). Agarose gel electrophoresis was performed to check if the target fragment was the only amplicon and if its size was close to the expected one. The amplicons obtained from the same plant were mixed in a single tube and submitted for NGS sequencing. Illumina sequencing was conducted in Genomed LLC (Moscow, Russia). The DNA libraries were prepared using Swift 2S™ Turbo DNA Library Kits. In the process of library preparation, the content of each tube, corresponding to a single plant, was labeled with an individual DNA barcode. The sequencing was performed on the MiSeq system. After de-barcoding, the results were obtained for each submitted test tube separately as two files of short paired-end reads. Further, the total sequences of the three genes for each *Aegilops tauschii* plant were reconstructed from the NGS data using the undermentioned algorithm.

### 4.4. Bioinformatic Treatment of the Sequencing Results

The quality of sequencing data was assessed using FastQC software. In general, the quality of the obtained reads was sufficient for further analysis. To assemble gene sequences from short reads, we made a chain of programs that operates on the principle of reference-assisted de novo assembly [46]: SPAdes 3.14.0 was used for assembling contigs with parameters set by default [47]. CAP3 was used for assembling contigs into supercontigs using a reference sequence [48]. Mild parameters for CAP3 were taken, allowing alignment of contigs to the reference even if substantial differences are present (match score factor m = 40, overlap percent identity cutoff p = 70, gap penalty factor g = 1). To make a consensus sequence from the CAP3 alignment after discarding the initial reference sequence, the Consensus program was written in Python (https://github.com/MikhailBazhenov/Consensus). The resulting de novo assembled sequences contained inaccuracies that were further corrected. For correction of inaccuracies, we used reads mapping, variant calling, consensus building and making consensus sequence as a new reference in 6 iterations. For this step, scalable nucleotide alignment program (SNAP) v1.0 was used for mapping the reads [49], SAMtools 1.10—for filtering unmapped reads and file format conversion, FreeBayes—a haplotype-based variant detector was used for variant calling with parameters set by default [50], Vcflib for filtering low-quality variants (https://github.com/vcflib/vcflib), BCFtools 1.10.2 [51]—for the introduction of alternative variants to the reference sequence (making consensus). Variants with a quality score of more than 20 (QUAL > 20) were used for making consensus sequences at each iteration and with allele balance more than 0.25 (AB > 0.25) at the last two iterations to remove random sequencing errors.

Thus, obtained sequences were aligned to each other, to the reference sequence of the AL8/78 *Aegilops tauschii* genome assembly, and to the common wheat homologous genes sequences from wheat genome assembly RefSeq1.0 using MEGA X software and the MUSCLE multiple sequence alignment algorithm [52,53]. The spans of exons and coding sequences were deduced by an alignment with annotated exons and protein-coding sequences for the *Aegilops tauschii* AL8/78 Aet_MR_1.0 genome assembly. The translation to an amino acid sequence was performed in GeneDoc.

The functional significance of amino acid substitutions was predicted using the PROVEAN online service (http://provean.jcvi.org). PROVEAN algorithm uses the change in the alignment score caused by an amino acid variation in the query protein sequence, which is aligned to the functional homolog sequences found in a database as a measure of the impact of the variation on the protein functionality. The more dissimilar to the functional homologs becomes the protein after the variation introduction, the lower is the delta score and the higher the negative impact on the biological activity is assumed. The PROVEAN algorithm consists of two steps. The first is the collection of homologous sequences for the supporting sequence set, and the second is the computation of an unbiased averaged delta score based on alignments of the query sequence with itself and the supporting sequence set. PROVEAN predictive ability was shown to be highly comparable with other leading tools used for the same purpose. PROVEAN score threshold of −2.282 for dividing deleterious variations (lower score) from neutral (higher score) gives prediction accuracy of about 77% for non-human proteins [32].

Entire sequences, including promoters, were used for evolutionary analyses of the genes. Evolutionary analyses were conducted in MEGA X [53] using the maximum-likelihood method and Hasegawa–Kishino–Yano model [54]. All positions containing gaps and missing data were eliminated. Bootstrap support values were calculated using 500 replicates. The trees were drawn to scale, with branch lengths measured in the number of substitutions per site. To root the trees, the homologs from wheat subgenome B were added as outgroups in evolutionary analyses.

### 4.5. Statistical Treatment

One-way analysis of variance was performed using Statistica 6.0 software.

## 5. Conclusions

In this work, we explored allelic diversity of the three genes—*Rht-D1*, *Gid1*, and *Gid2*—involved in the signaling pathway of gibberellins of the diploid grass species *Ae. tauschii*, one of the ancestral species of bread wheat and the donor of its D subgenome, and found a great number of polymorphisms, including those which lead to changes in the amino acid sequence of the encoded proteins. The RHT-D1 protein showed to have four isoforms (amino acid sequence variants), and the GID1 and GID2 proteins possess two isoforms each. Analysis of the coexistence of various isoforms of the three proteins showed that their combinations were not random in *Ae. tauschii* accessions, which may indicate the functional significance of their differences and the selectivity of certain combinations. However, we failed to show their relationship with plant height in the studied *Ae. tauschii* accessions. Existing polymorphism in plant height between accessions could be explained by spring or winter growth habit, that is, by the genes *Response to vernalization* (*Vrn*) [55]. The phenotypic expression of the discovered alleles and the expediency of their use in the modern breeding program of the bread wheat remains a question for future research. The new alleles found in this study may prove to be useful for fine plant growth regulation if transferred to widely cultivated wheat species.

## Figures and Tables

**Table 1:** Frequencies of the *Rht-D1* alleles within the studied collection of *Ae. tauschii* accessions and corresponding protein isoforms.

Allele	Frequency	Protein Isoform
*Rht-D1a_7*	0.42	B
*Rht-D1a_5*	0.33	A
*Rht-D1a_8*	0.08	A
*Rht-D1a_9*	0.04	A
*Rht-D1a_10*	0.04	C
*Rht-D1a_11*	0.04	B
*Rht-D1a_12*	0.04	D

Note: the frequencies of the isoforms are as follows: A—0.46; B—0.46; C—0.04; D—0.04.

**Table 2:** Amino acid variations in the RHT-D1 protein of *Ae. tauschii.*

Amino Acid Variations	Protein Isoforms				PROVEAN Score
	A	B	C	D	
T162V	−	+	−	+	0.294
G334S	+	−	−	−	−3.761 *
G622A	−	+	−	−	0.674

* significant amino acid replacement according to the PROVEAN forecast [32]. All amino acid variations are given relative to the RHT-D1 amino acid sequence of cv. Chinese Spring of common wheat. The presence of a certain variation is indicated by the “+” sign, and the absence is indicated by the “−” sign.

**Table 3:** Frequencies of the *Gid1-D* alleles within the studied collection of *Ae. tauschii* accessions and corresponding protein isoforms.

Allele	Frequency	Protein Isoform
*Gid1-D1a*	0.25	A
*Gid1-D1b*	0.15	B
*Gid1-D1c*	0.13	A
*Gid1-D1d*	0.08	A
*Gid1-D1e*	0.08	B
*Gid1-D1f*	0.06	A
*Gid1-D1g*	0.04	A
*Gid1-D1h*	0.04	A
*Gid1-D1i*	0.04	A
*Gid1-D1j*	0.04	A
*Gid1-D1k*	0.04	A
*Gid1-D1l*	0.02	B
*Gid1-D1m*	0.02	A

Note: the frequency of isoform A is 0.75; isoform B is 0.25.

**Table 4:** Frequencies of the *Gid2-D* alleles within the studied collection of *Ae. tauschii* accessions and corresponding protein isoforms.

Allele	Frequency	Protein Isoform
*Gid2-D1a*	0.33	A
*Gid2-D1b*	0.17	B
*Gid2-D1c*	0.13	A
*Gid2-D1d*	0.13	A
*Gid2-D1e*	0.13	A
*Gid2-D1f*	0.04	A
*Gid2-D1g*	0.04	A
*Gid2-D1h*	0.04	A

Note: the frequency of isoform A is 0.83; isoform B is 0.17.

**Table 5:** Joint finding of RHT-1, GID1, and GID2 protein isoforms in *Ae. tauschii* accessions.

		RHT-1				The *p* Value of Fischer’s Exact Test *
		A	B	C	D	
**GID1**	**A**	10	22	2	2	0.0001
	**B**	12	0	0	0	
**GID2**	**A**	14	22	2	2	0.0036
	**B**	8	0	0	0	

* Only isoforms A and B of the RHT-1 protein were taken for calculations.

**Table 6:** Joint finding of the GID1 and GID2 protein isoforms in *Ae. tauschii* accessions.

		GID1		The *p* Value of Fischer’s Exact Test
		A	B	
**GID2**	**A**	33	7	0.0166
	**B**	3	5	

## Supplementary Materials

The following are available online at https://www.mdpi.com/2223-7747/9/12/1696/s1, Table S1: The characteristics of the *Aegilops tauschii* Coss. accessions, including the genotypes for the three genes (*Rht-1*, *Gid-1*, *Gid-2*) and corresponding protein isoforms involved in the gibberellin hormone signal transduction pathway.
