# Supplementary material for: The Allelic Diversity of the Gibberellin Signaling Pathway Genes in Aegilops tauschii Coss
Source: Plants (Basel). 2020 Dec 2;9(12):1696. doi: 10.3390/plants9121696 (PMC7761575; doi:10.3390/plants9121696)
Supplement: Supplementary file 1 [file plants-09-01696-s001.zip › Table S1.pdf]

Table S1. The characteristics of the *Aegilops tauschii* Coss. accessions including the genotypes for the three genes (*Rht-1*, *Gid-1*, *Gid-2*) and corresponding protein isoforms involved in the gibberellin hormone signal transduction pathway

Accessions[1]	Subspecies (ssp.), variety (var.)	Country of origin	*Rht-1*		*Gid-1*		*Gid-2*		Plant height[4], cm
			Allele	Protein isoform	Allele	Protein isoform	Allele	Protein isoform	
K-1099	ssp. *tauschii*	Azerbaijan (Lerik District)	*Rht-D1a_5*	A	*Gid1-D1d*	A	*Gid2-D1f*	A	68±4
K-2271	ssp. *tauschii*	Armenia (Kotayk Province)	*Rht-D1a_5*	A	*Gid1-D1d*	A	*Gid2-D1h*	A	88±4
K-497	ssp. *strangulata*	Nagony Karabakh	*Rht-D1a_5*	A	*Gid1-D1h*	A	*Gid2-D1e*	A	75±7
C 21-5127	ssp. *strangulata*	Azerbaijan	*Rht-D1a_5*	A	*Gid1-D1b*	B	*Gid2-D1c*	A	n. d.
K-1216	ssp. *tauschii*	Georgia	*Rht-D1a_5*	A	*Gid1-D1b*	B	*Gid2-D1g*	A	65±0
KU-2074	ssp. *strangulata*	Iran	*Rht-D1a_5*	A	*Gid1-D1b*	B	*Gid2-D1c*	A	n. d.
K-1723	ssp. *tauschii*	Azerbaijan	*Rht-D1a_9*	A	*Gid1-D1b/e*[2]	B	*Gid2-D1b*	B	48±11
K-3187	ssp. *tauschii* var. *typica*	Armenia	*Rht-D1a_5*	A	*Gid1-D1e/f*	B/A	*Gid2-D1c*	A	98±4
K-1112	ssp. *tauschii*	Azerbaijan	*Rht-D1a_8*	A	*Gid1-D1e/f*	B/A	*Gid2-D1b*	B	58±4
K-4049	ssp. *tauschii*	Iran	*Rht-D1a_5*	A	*Gid1-D1e/f*	B/A	*Gid2-D1b*	B	70±7
K-527	ssp. *tauschii*	Armenia	*Rht-D1a_8*	A	*Gid1-D1l/m*	B/A	*Gid2-D1b*	B	68±4
C 21-4030	ssp. *tauschii*	Unknown	*Rht-D1a_7*	B	*Gid1-D1c*	A	*Gid2-D1a*	A	n. d.
C 21-5130	ssp. *tauschii*	Azerbaijan	*Rht-D1a_11*	B	*Gid1-D1j*	A	*Gid2-D1a*	A	n. d.
K-1657	ssp. *tauschii*	Pakistan	*Rht-D1a_7*	B	*Gid1-D1a*	A	*Gid2-D1a*	A	68±4
K-396	ssp. *tauschii*	Uzbekistan	*Rht-D1a_7*	B	*Gid1-D1g*	A	*Gid2-D1a*	A	78±4
K-428	ssp. *tauschii* var. *typica*	Turkmenistan	*Rht-D1a_7*	B	*Gid1-D1c*	A	*Gid2-D1a*	A	75±0
K-4564	ssp. *tauschii*	Syria	*Rht-D1a_7*	B	*Gid1-D1a*	A	*Gid2-D1a*	A	78±4
K-608	ssp. *tauschii* var. *meyeri*	Georgia	*Rht-D1a_7*	B	*Gid1-D1a*	A	*Gid2-D1d*	A	70±7
K-865	ssp. *tauschii*	Unknown	*Rht-D1a_7*	B	*Gid1-D1a*	A	*Gid2-D1d*	A	70±0
K-896	ssp. *tauschii*	Afghanistan	*Rht-D1a_7*	B	*Gid1-D1a*	A	*Gid2-D1d*	A	43±4
K-994	ssp. *tauschii*	Afghanistan	*Rht-D1a_7*	B	*Gid1-D1c*	A	*Gid2-D1a*	A	75±0
KT 120-13	ssp. *tauschii*	China	*Rht-D1a_7*	B	*Gid1-D1a*	A	*Gid2-D1a*	A	n. d.
KT 120-10	ssp. *tauschii*	China	*Rht-D1a_10*	C	*Gid1-D1i*	A	*Gid2-D1e*	A	n. d.
AL8/78[3]	ssp. *strangulata*	Armenia	*Rht-D1a_12*	D	*Gid1-D1k*	A	*Gid2-D1e*	A	n. d.

[1] The *Ae. tauschii* accessions partially were provided by the Federal Research Center Vavilov All-Russian Institute of Plant Genetic Resources (VIR), Saint-Petersburg, Russia (“K“); Czech Institute of Plant Industry, Praga-Ruzine, Czech Republic (“C 21”); Kyoto University, Kyoto, Japan (“KU“); Institute Biology of Kihara Foundation, Yokohama, Japan (“KT“). [2] Another allele or protein isoform is given after a slash if several of them are present. [3] The genotypes of AL8/78 were deduced from its genome assembly Aet_MR_1.0. [4] the standard deviation is given. n. d. – no data.
